# Undetected intracardiac thrombus and subsequent embolism following cardiopulmonary bypass: a case report

**DOI:** 10.1186/s13019-026-03861-z

**Published:** 2026-02-04

**Authors:** Atsuyuki Mitsuishi, Ren Saito, Naoki Edo, Yujiro Miura

**Affiliations:** Department of Cardiovascular Surgery, Kochi Medical School Hospital, 185-1, Kohasu, Nankoku-shi, 783-8505 Okohcho, Kochi Prefecture Japan

**Keywords:** Full heparinization, Intracardiac thrombus, Transesophageal echography, Embolization, Low ejection fraction, Left ventricular aneurysm, Cancer, Case report

## Abstract

**Background:**

Heparin is commonly administered to prevent thrombosis during cardiopulmonary bypass (CPB). However, the development of intracardiac thrombi under CPB has still been reported.

**Case summary:**

A 79-year-old man with three-vessel coronary artery disease and a history of bladder cancer underwent on-pump coronary artery bypass grafting. His cardiac function was compromised by a low ejection fraction. After aortic declamping, the arterial waveform in the right radial arterial line disappeared abruptly, and emergent thrombectomy was performed for thrombotic occlusion of the right radial artery. On the following day in the intensive care unit, the patient developed acute left-sided paralysis without any documented episode of atrial fibrillation. Computed tomography confirmed occlusion of the right internal carotid artery, prompting a second emergent thrombectomy.

**Discussion:**

Despite full heparinization, intracardiac thrombus formation can still occur during CPB due to patient- and surgery-related factors such as cancer, coronary artery disease, low ejection fraction, and cardiac arrest. While transesophageal echocardiography (TEE) is the gold standard for detecting intracardiac thrombi, it can yield false negatives.

**Conclusion:**

This highlights the importance of thorough TEE screening just before aortic declamping in patients at high risk for intracardiac thrombus, and possibly the need for additional imaging modalities to improve thrombus detection.

## Background

Heparin is routinely administered to prevent thrombosis during cardiopulmonary bypass (CPB). Nevertheless, the formation of intracardiac thrombi during CPB continues to be reported. Although transesophageal echocardiography (TEE) is widely used for detecting intracardiac thrombi, it can occasionally yield false negatives, underscoring the importance of careful evaluation just before aortic declamping in high-risk patients.

## Case presentation

**Fig. 1:**
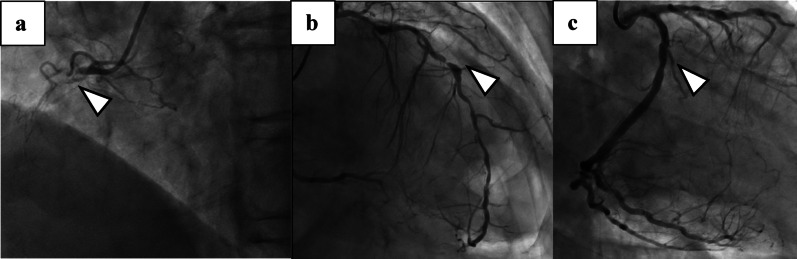
Preoperative coronary angiography.** a**: Proximal right coronary artery with 100% stenosis. **b**: Mid-left anterior descending artery with 90% stenosis. **c**: Proximal left circumflex artery branch with 100% stenosis

A 79-year-old man presented with dyspnea. Transthoracic echocardiography (TTE) demonstrated a reduced left ventricular (LV) ejection fraction of 29%, with regional wall motion abnormality predominantly in the inferoposterior territory, without evidence of valvular pathology, mural thrombus, or LV thrombus formation. Preoperative imaging also revealed an apical outpouching; however, diagnostic criteria for a true aneurysm were not met, as there was no discrete neck, no dyskinesis, no intracavitary thrombus, and only minimal geometric distortion. Accordingly, ventriculotomy was not indicated. Coronary angiography revealed a three-vessel lesion (Fig 1). Carotid ultrasound was performed preoperatively with no significant stenosis.

The patient received diuretics and β-blockers for acute heart failure. In addition, the patient had gross hematuria, which resulted in bladder tamponade. After the patient was referred to the urology department and diagnosed with bladder cancer (T2N0M0), he was recommended to undergo cardiovascular surgery for three-vessel disease.

**Fig. 2:**
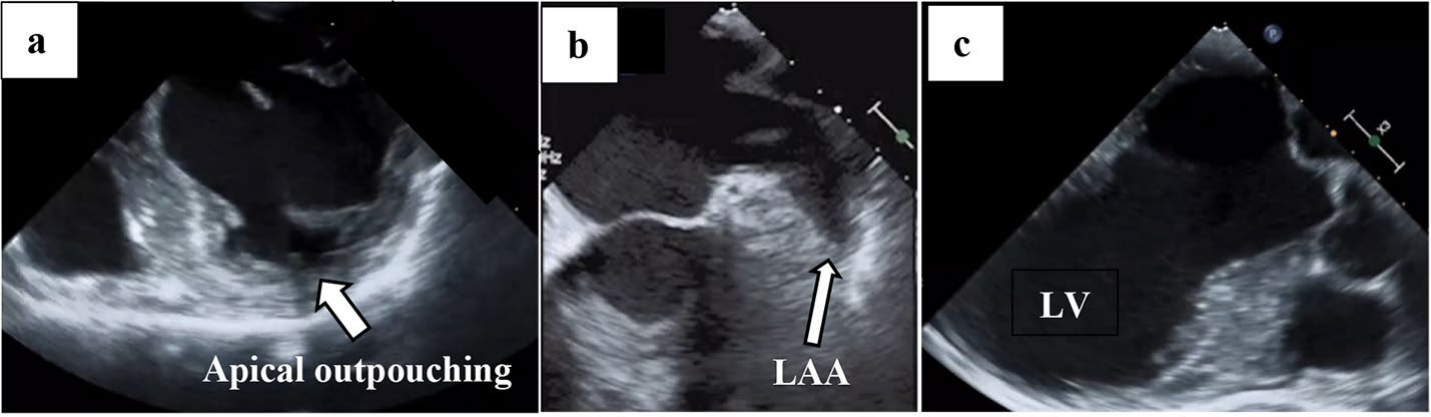
Intraoperative transesophageal echocardiography. **a** Apical outpouching of the left ventricle. **b** Left atrial appendage. **c** Left ventricle. LV: Left ventricle, LAA: Left atrial appendage.

**Fig. 3:**
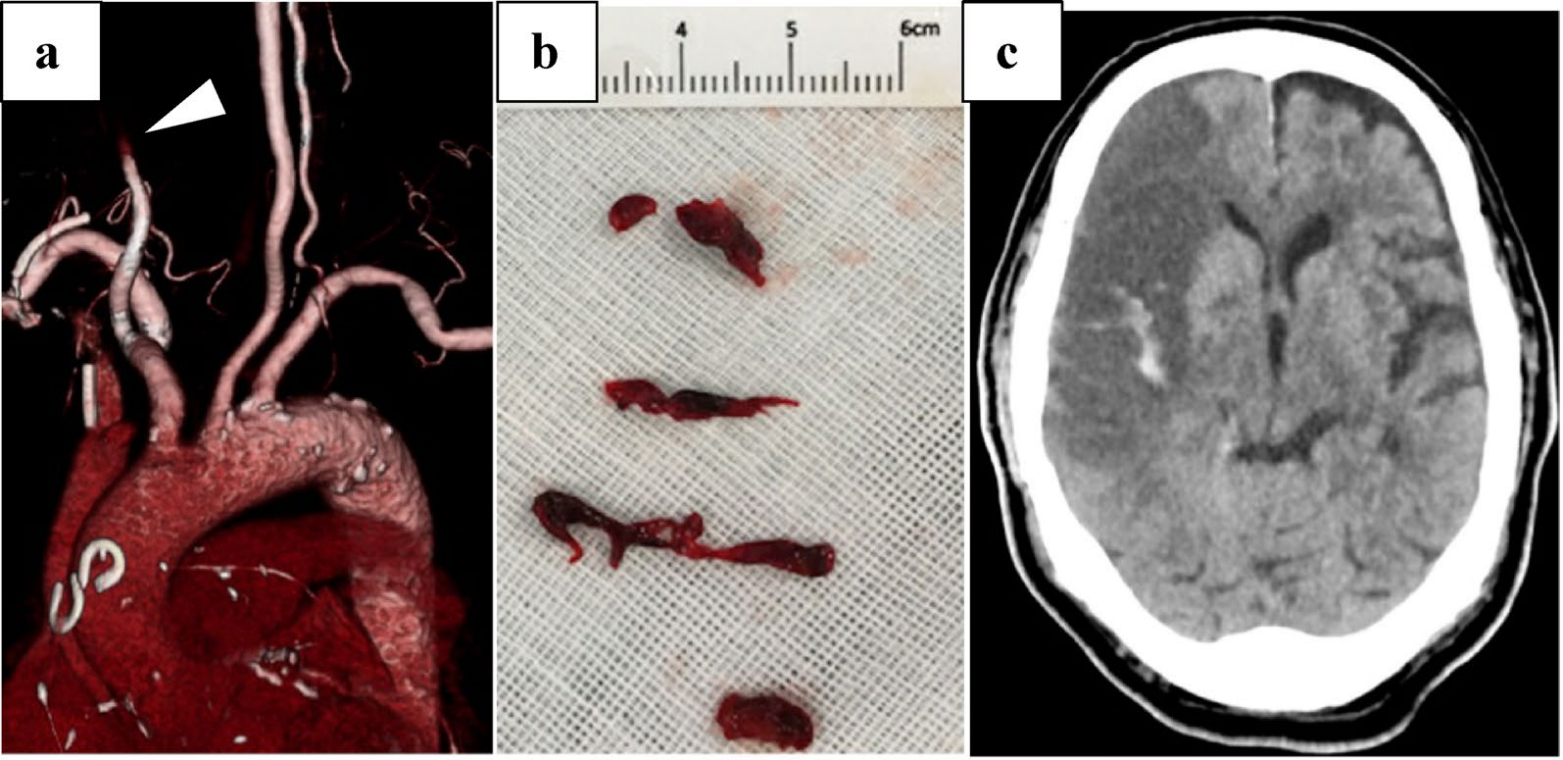
**a**: Right carotid embolization on computed tomography. **b**: Thrombus from the right carotid artery. **c**: Hemorrhagic brain infarction on computed tomography

A three-vessel on-pump coronary artery bypass grafting (CABG) was performed. CPB was established via median sternotomy using standard aortic and right atrial cannulation with antegrade cardioplegia under full heparinization (activated clotting time; ACT > 500 s). ACT was measured every 60 min and maintained above 500 s after CPB started. Coronary revascularization was achieved by grafting saphenous vein grafts to the posterior descending and lateral arteries and the left internal mammary artery to the left anterior descending artery. Cardiac arrest time and CPB time were 153 and 208 min, respectively. Cardiac arrest and CPB times were longer than average due to diffuse multivessel disease requiring careful distal target preparation and precise anastomosis, though the procedure proceeded without intraoperative complications. Before removal of the aortic vent, TEE was performed to assess the aortic, mitral, and tricuspid valves, as well as regional wall motion, and confirmed the absence of intracardiac thrombus (Fig. 2). Following aortic declamping, arterial pressure waves could not be detected through the right radial arterial line. After completion of the cardiac procedure, protamine was administered (ACT 133 s). The absence of arterial pressure tracing in the right radial line prompted bedside ultrasonographic evaluation, which confirmed thrombotic occlusion of the right radial artery. The patient was subsequently transferred to the hybrid operating room for emergency thrombectomy using a 4Fr Fogarty catheter and thrombus was retrieved. Post‑operative coagulation status in ICU was as follows: PT 15.4 s, PT% 52.0, INR 1.36, APTT 175.6 s, APTT% 19.3, fibrinogen 185 mg/dL. On postoperative day 1 (POD1), the patient was extubated; however, several hours later, he developed sudden left-sided hemiparalysis without any episode of atrial fibrillation. Computed tomography scan showed occlusion of the right internal carotid artery, and emergency thrombus removal was performed (Fig. 3).

**Fig. 4:**
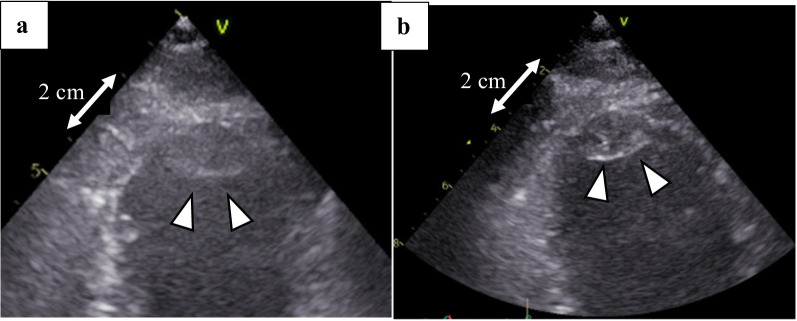
Postoperative echocardiography with intracardiac thrombus (white arrows). **a**: 7 days after the surgery. **b**: 1 month after surgery

Postoperative TTE revealed an intracardiac thrombus (21 × 11 mm). Laboratory results were as follows: heparin-induced thrombocytopenia antibody (−), lupus anticoagulant 0.97 (normal: <1.3), protein C 89% (normal: 64%–146%), and protein S 76% (normal: 67%–164%). Due to bleeding concerns, oral anticoagulant therapy with warfarin initiation (target international normalized ratio = 2–3) was delayed and started POD9. Histopathological analysis of both thrombi demonstrated acute fibrin-dominant clot without evidence of plaque, lamination, or organizational fibrosis, consistent with recent formation rather than chronic deposition. At 1-month follow-up TTE, the ejection fraction was 27% with severe hypokinesis of the inferoposterior wall (base–mid), and the size of the intramural thrombus decreased (Fig. 4). The patient who was ambulatory was transferred to another hospital 28 days postoperatively.

## Discussion

The technical challenge of preventing thrombosis within the extracorporeal circuit during CPB was addressed with heparin. Heparin rapidly promotes thrombin inhibition; however, it cannot completely prevent thrombin formation during CPB [1, 2].

In this case, two main causes of embolism after declamping during CPB are considered: an old thrombus created preoperatively and a new thrombus formed intraoperatively.

The former cause involves a pre-existing intracardiac thrombus that could not be detected by echocardiography. Although TEE is considered the gold standard for detecting intracardiac thrombus, it can yield false-negative results [3], particularly in the left atrial appendage [4] and also in the left ventricular apex [5], as seen in this case. Consequently, this thrombus could not have been detected by TTE preoperatively or by TEE intraoperatively. The American Society of Echocardiography guidelines [6] state that comprehensive epicardial echocardiography (EpE) can be performed efficiently and safely, and may be superior to TEE as it provides higher image resolution when using high-frequency probes. Awasthy et al. [7] also reported that EpE allows the surgeon to direct the views to interrogate specific areas of concern. However, it remains uncertain whether EpE would have detected the intracardiac thrombus in this case.

The latter cause involves rapidly formed thrombi during CPB. Despite heparin use, thrombin formation is not completely prevented, and several case reports have described rapid thrombus formation during CPB [1, 2]. These causes can be categorized as patient-related and/or surgery-related (Table 1). Patient-related factors include blood and cardiac abnormalities. In this case, the presence of cancer created a procoagulant state, increasing the risk of intracardiac thrombus [8]. In addition, patients with coronary artery disease are at higher risk of intramural thrombus [9]. However, no causative blood abnormality was found in this patient, and there was no evidence of massive or multiple clot formation in the intracavity [10] or CPB circuit [11], suggesting no significant blood abnormality. Regarding cardiac abnormalities, patients with low ejection fraction are more likely to develop intracardiac thrombi [12, 13]. Blood stasis in an aneurysmal site can further promote mural thrombus formation and the risk of embolization [14].

Surgery-related factors include surgical trauma, such as vent tube insertion [15] and cardiac arrest [16], which induces an ultimate low-flow state that can promote LV thrombus formation.

Venting was adequate overall, although intermittent suboptimal drainage cannot be excluded. Therefore, in this case, blood stasis in the LV and ventricular wall pathology may have contributed to the formation of an intracardiac thrombus.

In summary, considering that intracardiac thrombi are often found incidentally just before aortic declamping [1, 2], regardless of whether the thrombus was pre-existing or formed intraoperatively, and irrespective of patient- or surgery-related mechanisms, these observations highlight the importance of meticulous intraoperative assessment and suggest a potential role for routine screening with TEE supplemented by EpE just before aortic declamping, particularly in patients with underlying risk factors. Although similar cases have been reported, they remain rare. Further reports are needed to strengthen the evidence base.

## Conclusion

This case illustrates that despite the routine use of heparin during CPB, thrombin formation and subsequent intracardiac thrombus formation may still occur. Although TEE remains the gold standard for intraoperative thrombus detection, thrombi in anatomically recessed regions such as the LV apex may be missed. These findings suggest that TEE assessment immediately prior to aortic declamping, and in selected cases supplementary imaging such as EpE may be considered for patients at elevated thrombotic risk. Further accumulation of clinical experience is required before this approach can be recommended as a routine practice.

**Table 1:** Factors associated with intracardiac thrombus formation

Factor type	Specific factors	Reference
Patient-related	Cancer	[8]
	Coronary artery disease	[9]
	Blood abnormality	[10, 11]
	Heparin-induced thrombocytopenia antibody	
	Lupus anticoagulant	
	Protein C deficiency	
	Protein S deficiency	
	Low ejection fraction	[12, 13]
	Blood stasis in an aneurysmal site	[14]
Surgery-related	Surgical trauma such as vent tube insertion	[15]
	Cardiac arrest	[16]

## Data Availability

All data generated or analyzed during this study are included in this article.

## Abbreviations

CPB: Cardiopulmonary bypass
TEE: Transesophageal echocardiography
TTE: Transthoracic echocardiography
EpE: Epicardial echocardiography
